# Early diagnosis and prognostic prediction of secondary bloodstream infections caused by *Acinetobacter baumannii* in critically ill patients *by* machine-learning algorithms

**DOI:** 10.3389/fcimb.2025.1667176

**Published:** 2026-01-08

**Authors:** Hengxin Chen, Wenjia Gan, Xianling Zhou, Pingjuan Liu, Tangdan Ding, Hongxu Xu, Peisong Chen, Yili Chen

**Affiliations:** 1 Department of Laboratory Medicine, The First Affiliated Hospital of Sun Yat-sen University, Guangzhou, Guangdong, China; 2 Department of Clinical Immunology, The Third Affiliated Hospital of Sun Yat-sen University, Guangzhou, Guangdong, China

**Keywords:** *Acinetobacter baumannii*, diagnosis, machine learning, prognosis, secondary bloodstream infections

## Abstract

**Background:**

Secondary bloodstream infections (sBSI) caused by *Acinetobacter baumannii* (AB) are a major threat to patient safety in the Intensive Care Unit (ICU) due to their prevalence and severity. Developing accurate predictive models is crucial for enhancing clinical decision-making and improving patient outcomes. This study aimed to leverage machine learning (ML) to create a diagnostic model for predicting the risk of AB-sBSI in ICU patients and a prognostic model for assessing the associated 30-day mortality risk.

**Methods:**

The multicenter, retrospective study enrolled 4,267 ICU patients with *AB* isolated from non-blood sites. Of these, 337 patients developed bloodstream infection. The analysis included 70 patients with confirmed *AB* secondary bloodstream infection (AB-sBSI) and 76 age and sex matched controls with non AB-sBSI. For 30-day mortality assessment, the AB-sBSI patients were categorized into non-survivors (n=39) and survivors (n=31). Demographic, microbiological, and laboratory data encompassing hematological, coagulation, and inflammatory markers were analyzed. Fourteen machine learning models were evaluated using the Deepwise and Beckman Coulter DxAI platforms with five-fold cross-validation. Model performance was assessed using five standard metrics, and the DeLong test was applied for AUC comparison. After data preprocessing, patients were enrolled to form an external validation cohort.

**Results:**

The AB-sBSI risk diagnosis model, constructed with 11 features, identified red cell distribution width as the most significant predictor. The AdaBoost model outperformed both comparative models (Linear Discriminant Analysis, Logistic Regression, LinearSVC) and the conventional biomarker C-reactive protein (AUC = 0.66), with AUCs of 0.937 in training and 0.786 in validation. For 30-day mortality prediction, another model based on 11 features selected lymphocyte count as the most influential variable. The AdaBoost model showed prominent efficacy, surpassing other model (Multilayer Perceptron, BernoulliNB, SGD) and achieving AUC values of 0.986 in training and 0.821 in validation.

**Conclusion:**

We developed two ML based models for predicting AB-sBSI risk and 30-day mortality. As a preliminary exploration, both models have been converted into accessible web tools. These tools are designed to assist clinicians in making informed decisions and promptly adjusting treatment strategies for critically ill patients.

## 1 Background

*Acinetobacter baumannii* (AB) poses a significant threat in the ICU setting, particularly due to its association with severe infections and high mortality rates among critically ill patients (Harding et al., 2018). The mortality rate among ICU patients with AB-associated bloodstream infection(AB-BSI) ranges from 34% to 43.4% (Li et al., 2024; Yehya et al., 2025). With the increasing severity of multi-drug resistance and the dwindling options for effective medications, treating AB-BSI has become highly challenging (Doi, 2019). The early diagnosis and treatment of bloodstream infection (BSI) patients are essential to their prognosis, as timely and effective management of the infection can significantly improve outcomes (Timsit et al., 2020).

In critically ill patients, AB-BSI is frequently secondary to the isolation of the same bacterial strain from other body sites, such as pulmonary, intravenous catheter, and abdominal infections, a consequence of the patients’ compromised immune systems (Wisplinghoff et al., 2000; Rodríguez-Baño et al., 2004; Trottier et al., 2007). Consequently, effective diagnostic tools for predicting the risk of AB-associated secondary bloodstream infection (AB-sBSI) and for assessing its prognosis in ICU patients remain lacking. As the use of a single marker offers limited value in the diagnosis of BSI, according to some studies, the simple addition of new biomarkers on the basis of commonly used clinical diagnostic indicators can improve the diagnostic performance to some extent, however this approach does not fully resolve the challenges encountered in clinical practice (Taneja et al., 2017).

Leveraging the power of big data, machine learning (ML) has seen significant advancements (Peiffer-Smadja et al., 2020). These models have demonstrated particular potential in infectious diseases by aiding in the diagnosis of BSI. Models based on ML show high accuracy (AUROCs 0.874-0.94) in predicting BSI, outperforming traditional methods (Liang et al., 2024). Applications include forecasting carbapenem-resistant gram-negative bacteria and early detection in critically ill children (Han et al., 2024).

Beyond diagnosis, ML is increasingly applied in ICU settings to predict critical outcomes such as mortality, readmission, length of stay, and the risk of sepsis or acute respiratory distress syndrome (Sun et al., 2023). While these advances are promising, most existing BSI prediction studies are not specifically focused on secondary BSI caused by AB in ICU, thus leaving a critical gap. The development of dedicated models for AB-sBSI and their subsequent validation through multicenter prospective studies are therefore urgently needed (Ni et al., 2022; Xu et al., 2022).

To fill this gap, the aim of this study is to construct, based on machine learning algorithms, a risk prediction model for AB-sBSI in clinical ICU patients, as well as a model for assessing the prognosis of AB-sBSI.

## 2 Materials and methods

### 2.1 Study population and design

A multicenter, retrospective study was conducted between 2014 and 2021 across several institutions in Guangdong Province, China. The participating hospitals included the First Affiliated Hospital of Sun Yat-sen University, Ganzhou Hospital affiliated with Guangdong Provincial People’s Hospital, Heyuan People’s Hospital, Gaoming District People’s Hospital in Foshan City, and Huizhou Central People’s Hospital. Ethical approval for this research was obtained from the Ethics Committee of the First Affiliated Hospital of Sun Yat-sen University. Patients from the Nansha Branch of The First Affiliated Hospital of Sun Yat-sen University, admitted between July 2023 and June 2025, were enrolled to form an external validation cohort, including 18 non-AB-sBSI and 8 AB-sBSI cases.

**Figure 1:**
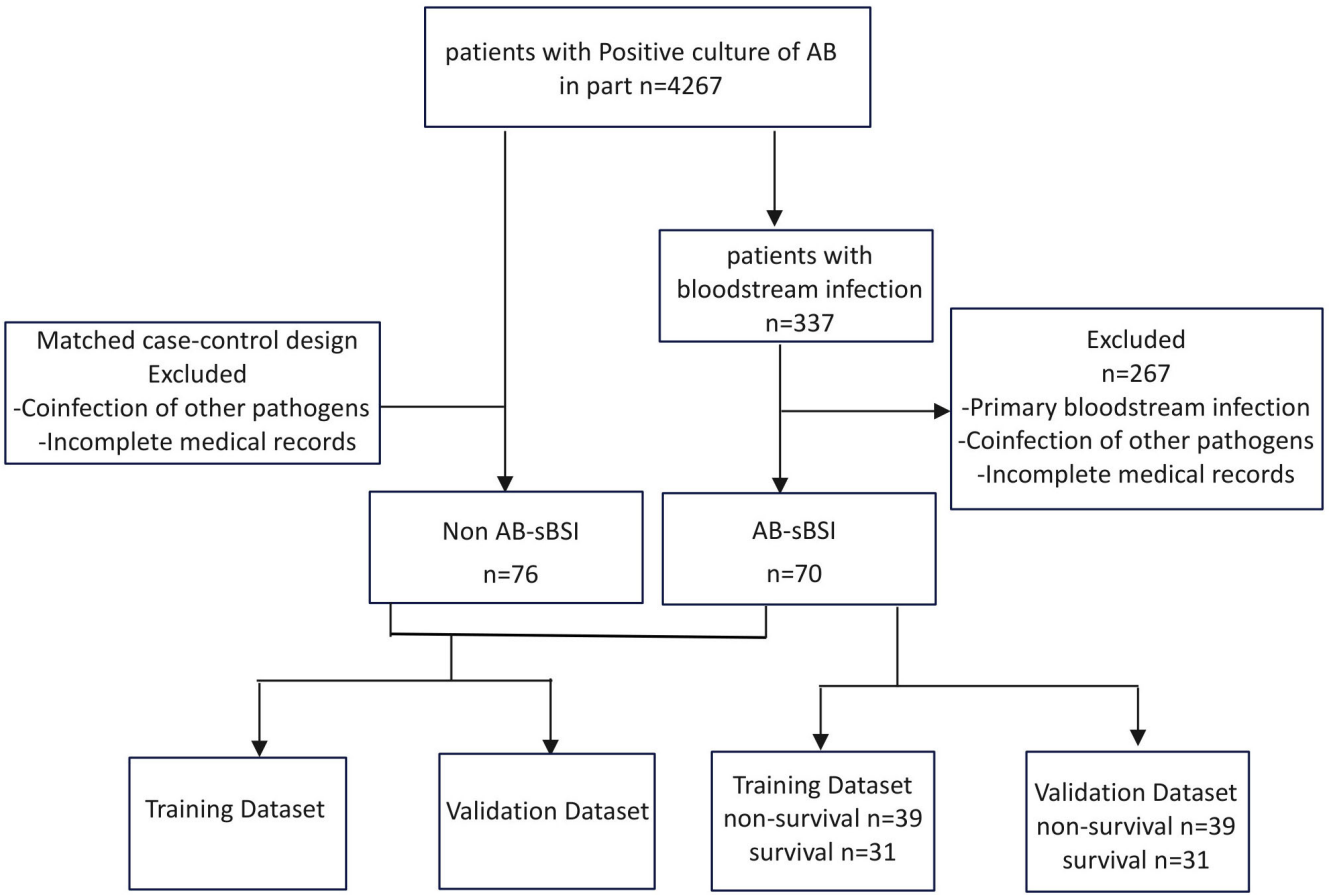
Data-cohort-workflow.

AS shown in **Figure 1**, a total of 4,267 intensive care unit (ICU) patients with *Acinetobacter baumannii* (AB) isolated from non-blood sites (e.g., respiratory tract, urinary tract, skin, and soft tissues) were initially screened. Of these, 337 ICU patients subsequently developed bloodstream infection (BSI). The final study cohort consisted of 70 patients with AB-associated secondary BSI (AB-sBSI) and 76 matched controls with non-AB-sBSI, matched based on age and sex (Mu et al., 2022).

The exclusion criteria were as follows: (a) incomplete or missing patient data; (b) coinfection with other pathogenic microorganisms; (c) patients whose first AB-positive culture was obtained from bloodstream; (d) for patients with AB detected in multiple sample cultures, only the first episode was included to ensure data independence. Furthermore, for prognostic analysis, the AB-sBSI group was stratified into survival and non-survival subgroups according to 30-day mortality.

The term “patients with coinfections by other pathogens” in our exclusion criteria refers specifically to patients with concurrent, active, culture-proven infections at a site other than the bloodstream, which were diagnosed by the treating physicians based on a combination of clinical symptoms, radiological evidence, and microbiological culture results.

### 2.2 Definition of bloodstream infection and secondary BSI

The diagnosis of bloodstream infection (BSI) required the presence of at least one positive blood culture, accompanied by two or more systemic signs of infection (including fever [>38°C], hypothermia [<36°C], tachycardia [heart rate >90/min], tachypnea [respiratory rate >20/min], leukocytosis [>12×10^9^/L], or leukopenia [<4×10^9^/L]) (Centers for Disease Control and Prevention, 2024).

A secondary BSI caused by Acinetobacter baumannii (AB-sBSI) was strictly defined as a BSI episode in which:

1. The same strain of AB was isolated from both a non-sterile site (e.g., respiratory tract, urinary tract) and a subsequent blood culture.
2. The non-blood site was clinically confirmed as the primary source of infection.
3. Primary BSI (i.e., without an identified non-blood source) and co-infections with other pathogens were excluded.

All suspected AB-sBSI cases were independently adjudicated by three senior intensive care physicians. To ensure diagnostic consistency, the physicians based their judgment on a pre-specified checklist incorporating the clinical, microbiological, and radiological criteria outlined above. The Prediction Starting Point (t=0) is definitively set as the time of first AB isolation from other site. The date of BSI onset was defined as the collection date of the first positive blood culture. The principle of the Secondary BSI Attribution Period was applied. All-cause mortality within 30 days following the first positive blood culture was defined as the primary outcome for prognostic analysis (Kontula et al., 2021).

### 2.3 Data collection

Clinical parameters of patients were obtained from electronic medical records. We recorded demographic data, APACHE II score, underlying diseases, and previous treatments. Additionally, microbiological data, ICU and hospital stay lengths, laboratory test results (hematological, coagulation indices, and inflammatory markers), and 30-day mortality were collected. Day of ICU hospitalization was indeed defined and collected as the time from ICU admission up until the time of first AB isolation from other site.

### 2.4 Isolation and identification of *Acinetobacter baumannii* strains

Bacterial culture was conducted according to the process of inoculation of clinical microbial specimens after specimen collection. Among them, blood culture was performed by BacT/Alert 3D system (bioMérieux, Marcyl Etoile, France). Species identification was completed using matrix-assisted laser desorption ionization–time-of-flight mass spectrometry (MALDI-TOF MS) (bioMé rieux, Marcy Etoile, France).

### 2.5 Antimicrobial susceptibility testing

Antimicrobial susceptibilities for isolates were determined with Vitek system (bioMérieux, Marcy l’Etoile, France). Susceptibility testing results were interpreted under the criteria recommended by the Clinical and Laboratory Standards Institute (CLSI, 2024). The quality control strain for susceptibility testing was *Escherichia coli* ATCC 25922 and *K. pneumoniae* ATCC 700603. Susceptibility testing results have been added to **Supplementary Tables S1** and **S2**.

### 2.6 Statistical analysis

Data analysis was performed using SPSS 25.0 software (IBM Corp, Armonk, NY, USA) and Deepwise platform. Data are presented as frequencies (percentages) for categorical variables and mean ± SD or median (IQR) for continuous variables. Group comparisons utilized Student’s t-test, the Wilcoxon test, or the Pearson χ² test. Cox regression was applied to compare survivors and non-survivors among patients with AB-sBSI. The Delong test was employed to compare the AUC values of different models. P-value < 0.05 was defined as the threshold for statistical significant.

### 2.7 Feature screening

The initial feature set was refined through a structured screening process prior to modeling. First, data quality filtering was applied: variables with more than 40% missing data were excluded (Gong et al., 2023; Rockenschaub et al., 2025). C -reactive protein (CRP) and C -reactive protein and albumin ratio (CAR) were removed in model for predicting the risk of AB-sBSI in ICU patients. Subsequently, univariate analysis was performed using Student’s t-test, the Wilcoxon test (for continuous variables), or the Pearson χ² test (for categorical variables), retaining variables with a p-value < 0.1. To address multicollinearity, a correlation analysis was conducted, removing one feature from any pair with a correlation coefficient > 0.9. L1-based feature selection method was then applied to further refine the predictor set. Finally, selections were reviewed for clinical applicability and cost-effectiveness. RDW was retained as previous studies have shown is an independent risk factor for predicting BSI.

### 2.8 Model training and validation

We used Deepwise & Beckman Coulter DxAI platform(https://dxonline.deepwise.com) was used as statistical analysis tool for this study. It used well-established python pyradiomics (version 3.0.1) and scikit-learn (version 0.22) package. In this retrospective cohort study, model training employed 5-fold cross-validation. To ensure a rigorous performance assessment, all feature selection was conducted independently within each training fold, and the corresponding test sets were used solely for evaluation to prevent data leakage. Repeat the loop n times and report the highest metric and its hyperparameter from the validation set. Finally, 11 features were used in the construction of the AB-sBSI mortality risk prediction model and the AB-sBSI 30-days mortality risk prediction model. The area under the curveReceiver operating characteristic (AUROC) curve analysis was done to evaluate the performance of each model. The AdaBoost model was finally selected based on its superior performance. Hyperparameter of the top four performing models were showed in **Supplementary Tables S3** and **S4**. The performance of each model was elucidated and compared using metrics such as the area under the curve (AUC), sensitivity, specificity, positive predictive value, and negative predictive value (Chen et al., 2024).

### 2.9 Features included in the modelling phase

The diagnostic model for predicting AB-sBSI risk in ICU patients comprised multiple clinically relevant variables, including red cell distribution width (RDW), day of ICU hospitalization, APACHE II score, presence of tracheal intubation or tracheostomy, use of carbapenems and antifungal medications, monocyte count (MONO), mean platelet volume (MPV), MPV to platelet count ratio (MPV/PC), D-dimer (D-D), and the type of inpatient ward.

The prognostic model for assessing 30-day mortality risk incorporated the following variables: total and ICU length of stay (LOS), APACHE II score, inflammatory markers (neutrophil-to-lymphocyte ratio (NLR), lymphocyte count (LY), monocyte count (MONO), procalcitonin (PCT), platelet parameters (MPV, MPV/PC, platelet count (PLT)), and most critically, the time interval between the initial isolation of AB from non-blood sites and subsequent BSI.

## 3 Results

### 3.1 AB-sBSI risk prediction diagnostic model

#### 3.1.1 Demographic and clinical characteristics

As shown in **Table 1**, patients who developed AB-sBSI were characterized by longer ICU hospitalization (19.000 *vs*. 13.000, p=0.010), higher APACHE Ⅱ score (19.000 *vs*. 15.000, p=0.003). Furthermore, they were recorded more frequently tracheal intubation/tracheotomy (94.3% *vs* 63.2%, p=0.000) and the use of carbapenems (72.9% *vs* 53.9%, p=0.018) and antifungal (65.7% *vs* 30.3%, p=0.000). In addition, the results of laboratory examination showed that the MONO count (0.551 ± 0.379 *vs* 0.685 ± 0.347, p=0.045), PLT count (162.005 ± 109.138 *vs* 229.073 ± 138.142, p=0.002), MPV (11.900 *vs* 10.630, p=0.001), MPV/PC (0.080 *vs* 0.050, p=0.002), D-dimer (D-D) (7.890 *vs* 4.060, p=0.002), CRP (101.246 ± 66.237 *vs* 68.841 ± 66.237, p=0.016) and CAR (3.509 ± 1.933 *vs* 2.385 ± 1.811, p=0.039) were significant differences between two groups. Notably, it was shown that the mortality rate was also higher (55.7% *vs* 27.6%, p=0.001).

**Table 1:** Baseline characteristics.

Variables	Non AB-sBSI (*n* = 76)	AB-sBSI (*n* = 70)	*p-*value
Basic features			
Male	52 (68.4%)	54 (77.1%)	0.238
Age (year)	59.158 ± 19.835	53.986 ± 19.491	0.115
Day of ICU hospitalization (day)	13.000(7.000-21.750)	19.000(9.500-42.250)	0.010 ^**^
APACHE Ⅱ score	15.000(11.000-20.500)	19.000(16.000-22.250)	0.003 ^**^
The inpatient ward			
EICU	6 (7.9%)	1 (1.4%)	0.150
MICU	10 (13.2%)	25 (35.7%)	0.001 ^**^
NICU	9 (11.8%)	4 (5.7%)	0.194
RICU	1 (1.3%)	3 (4.3%)	0.555
SICU	26 (34.2%)	26 (37.1%)	0.712
Tracheal intubation/tracheotomy	48 (63.2%)	66 (94.3%)	<0.001^**^
Usage of Antibiotics			
Carbapenems	41 (53.9%)	51 (72.9%)	0.018^*^
Antifungals	23 (30.3%)	46 (65.7%)	0.000^**^
Laboratory findings			
WBC (x10^9^/L)	11.837 ± 5.747	12.548 ± 8.364	0.549
NEU (x10^9^/L)	8.110(6.055-12.262)	8.800(6.497-13.095)	0.465
LY (x10^9^/L)	1.049 ± 0.583	0.931 ± 0.555	0.224
MONO (x10^9^/L)	0.685 ± 0.347	0.551 ± 0.379	0.045 ^*^
RDW (%)	0.170(0.150-0.190)	0.170(0.152-0.190)	0.626
PLT (x10^9^/L)	229.073 ± 138.142	162.005 ± 109.138	0.002 ^**^
MPV (fl)	10.630(9.713-11.665)	11.900(10.500-12.900)	0.001 ^**^
MPV/PC (fl/10^9^/L)	0.050(0.030-0.080)	0.080(0.048-0.128)	0.002 ^**^
PT (s)	14.680(13.230-16.800)	14.275(12.700-16.000)	0.321
INR	1.230(1.107-1.460)	1.233(1.093-1.365)	0.821
APTT (s)	39.659 ± 12.217	39.952 ± 11.103	0.883
TT (s)	16.965(15.575-18.385)	17.280(16.000-19.155)	0.153
FIB (g/L)	3.500(2.610-4.250)	2.990(2.127-4.240)	0.335
D-D (mg/L FEU)	4.060(1.870-8.470)	7.890(4.285-18.027)	0.002 ^**^
CRP (mg/L)	68.841 ± 54.389	101.246 ± 66.237	0.016 ^*^
ALB (g/L)	34.425(30.170-37.550)	33.815(29.402-36.250)	0.243
CAR (x10^-3^)	2.385 ± 1.811	3.509 ± 1.933	0.039 ^*^
PCT (ng/mL)	7.449 ± 13.681	9.070 ± 17.008	0.553
30-days survival	55 (72.4%)	31 (44.3%)	0.001 ^**^
Other time node			
Days from Tracheal intubation to the first isolation of AB in non-blood site (day)	6.000(1.000-11.000)	5.000(1.250-9.000)	0.552
carbapenem resistant AB	54(71.1%)	48(68.6%)	0.857

*P<0.05;**P<0.01.

APACHE II, The Acute Physiology and Chronic Health Evaluation II; WBC, leucocyte count; NEU, Neutrophil count; LY, Lymphocyte count; MONO, Monocyte count; RDW, Erythrocyte hemoglobin distribution width; PLT, platelet count; MPV, mean platelet volume; MPV/PC, mean platelet volume/platelet count; PT, prothrombin time; INR, international normalized ratio; APTT, activated partial thromboplastin time; TT, thrombin time; FIB, fibrinogen; D-D, D-dimer; CRP, C reactive protein; ALB, albumin; CAR,C reactive protein/albumin; PCT, procalcitonin; AB, *Acinetobacter baumannii.*

#### 3.1.2 Model building and evaluation

**Figure 2:**
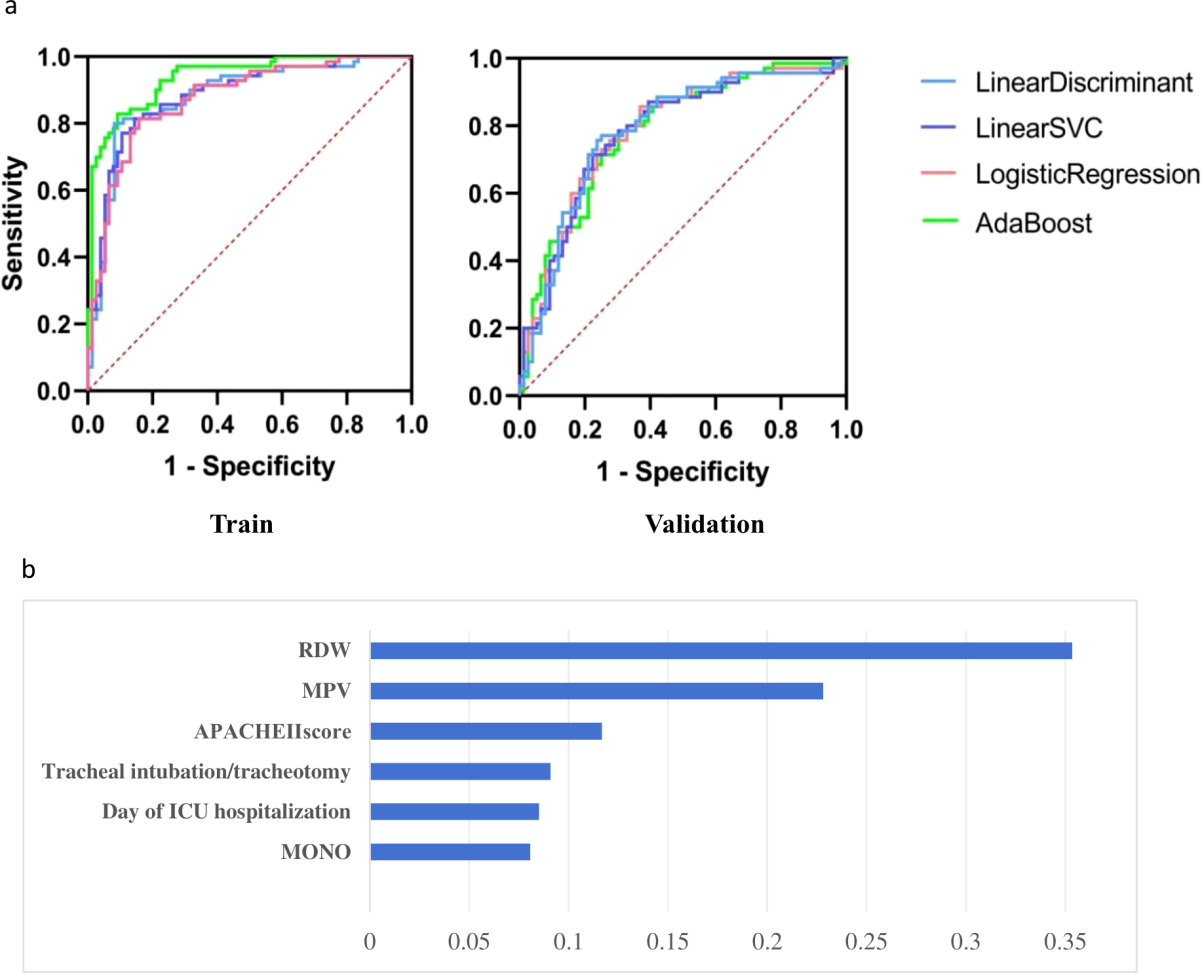
APACHE Ⅱ, The Acute Physiology and Chronic Health Evaluation II; RDW, Erythrocyte hemoglobin distribution width; MPV, mean platelet volume; MONO, Monocyte count. **(A)** The AUROC of Prediction performance in the train and validation datasets. **(B)** Feature Importance in AdaBoost model.

Eleven features were chosen as the input variables in our ML model to predict AB-sBSI risk. We compared 14 distinct algorithms to ascertain which one exhibited the most satisfying performance for our datasets. **Table 2** and **Figure 2a** illustrated the predictive capabilities of the top four models for AB-sBSI. Considering the AdaBoost model’s superior AUC in the training set and its statistically significant advantage over the other four models (p < 0.01, **Table 3**), we decided to adopt the AdaBoost model as our predictive algorithm. The model yielded AUROC results of 0.937 on the training dataset and 0.786 on the validation dataset, along with satisfactory specificity and sensitivity in the training dataset (0.908 and 0.814, respectively). Confusion matrix of AdaBoost model was in the **Supplementary Table S5**. Decision Analysis and Calibration plots of the AB-sBSI Risk Prediction Diagnostic Model was in the **Supplementary Materials 3**. The web application of the model can be access in https://dxonline.deepwise.com/prediction/index.html?baseUrl=%2Fapi%2F&id=49464&topicName=undefined&from=share&platformType=wisdom. A screenshot of the webpage was shown in **Supplementary Image 1**. Upon inputting the required parameters, the system can determine the likelihood of a patient having either AB-sBSI or Non AB-sBSI.

**Table 2:** Prediction performance of AB-sBSI using different ML models.

Dataset	ML models	AUROC	Sensitivity	Specificity	Negative predictive value	Positive predictive value
Train dataset N=146	AdaBoost	0.937 (0.900-0.974)	0.814 (0.708-0.888)	0.908 (0.822-0.955)	0.842 (0.752-0.904)	0.891 (0.802-0.944)
	LinearDiscriminantAnalysis	0.882 (0.816-0.934)	0.814 (0.712-0.888)	0.776 (0.668-0.857)	0.820 (0.722-0.890)	0.770 (0.664-0.852)
	LogisticRegression	0.873 (0.817-0.929)	0.814 (0.712-0.888)	0.803 (0.697-0.881)	0.824 (0.726-0.894)	0.792 (0.685-0.872)
	LinearSVC	0.884 (0.832-0.937)	0.857 (0.761-0.921)	0.776 (0.668-0.857)	0.855 (0.760-0.918)	0.779 (0.674-0.859)
Validation dataset N=146	LogisticRegression	0.787 (0.712-0.862)	0.757 (0.648-0.842)	0.724 (0.617-0.810)	0.764 (0.656-0.847)	0.716 (0.608-0.804)
	AdaBoost	0.786 (0.711-0.860)	0.686 (0.571-0.782)	0.750 (0.643-0.835)	0.722 (0.619-0.806)	0.716 (0.604-0.808)
	LinearDiscriminantAnalysis	0.786 (0.710-0.863)	0.771 (0.664-0.852)	0.737 (0.630-0.823)	0.778 (0.671-0.859)	0.730 (0.623-0.816)
	LinearSVC	0.782 (0.706-0.859)	0.743 (0.630-0.833)	0.711 (0.604-0.799)	0.750 (0.643-0.835)	0.703 (0.587-0.798)

**Table 3:** Delong test between four models in diagnostic performance.

Delong test between four models	Training delong		Validation delong	
Models	z	p	z	p
LinearDiscriminantAnalysis-AdaBoost	-2.866	0.004 **	0.023	0.982
AdaBoost-LogisticRegression	3.111	0.002 **	-0.073	0.942
LinearSVC-AdaBoost	-2.642	0.008 **	-0.135	0.893
LinearSVC-LinearDiscriminantAnalysis	1.576	0.115	-0.365	0.715
LinearSVC-LogisticRegression	1.734	0.083	-0.419	0.675
LinearDiscriminantAnalysis-LogisticRegression	0.306	0.76	-0.117	0.907

**p<0.01.

#### 3.1.3 Clinical features importance

Eleven features served as predictors in the AdaBoost model. **Figure 2b** highlights the top six laboratory tests with the highest significance features within the AdaBoost model. Each of these six tests had an importance value exceeding 0.05, with RDW holding the most substantial weight. This was followed by MPV, APACHE II score, the presence of tracheal intubation/tracheotomy, and MONO, ranked by their significance.

#### 3.1.4 Comparison of the AdaBoost model with CRP

**Figure 3:**
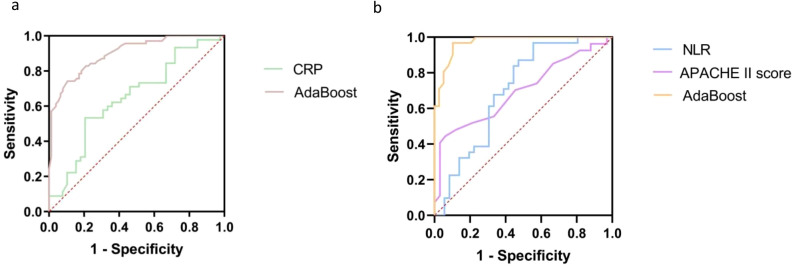
CRP, C reactive protein; APACHE Ⅱ, The Acute Physiology and Chronic Health Evaluation II; NLR, neutrophil-to-lymphocyte ratio. **(A)** The area under the receiver operating characteristic curves for the comparison of the AdaBoost prediction model with CRP. **(B)** The area under the receiver operating characteristic curves for the comparison of the AdaBoost prognosis model with APACHE Ⅱ and NLR.

We conducted a comparative analysis of the predictive performance between the AdaBoost model and the use of CRP alone, which is one of the most prevalent biomarkers for BSI in clinical practice (as shown in **Figure 3a**) (Lai et al., 2025). The findings revealed that the AdaBoost model outperformed CRP (AUROC 0.904 *vs*. AUROC 0.660).

#### 3.1.5 Interlabortary external validation

After data preprocessing, patients from the Nansha Branch of The First Affiliated Hospital of Sun Yat-sen University, admitted between July 2023 and June 2025, were enrolled to form an external validation cohort, including 18 non-AB-sBSI and 8 AB-sBSI cases. The performance of the AdaBoost prediction model on this external validation set is provided in **Supplementary Tables S6–S8**.

### 3.2 AB-sBSI prognostic prediction model

#### 3.2.1 Demographic and clinical characteristics

As reported in **Table 4**, the baseline characteristics of non-survival and survival groups in patients with AB-sBSI showed that non-survival group based on 30-days mortality was more associated with shorter hospitalization (18.000 *vs* 59.000, p=0.000), shorter ICU hospitalization (14.000 *vs* 37.000 days, p=0.009) and higher APACHE Ⅱ score (20.758 ± 4.555 *vs* 16.815 ± 5.664 points, p=0.004). Routine blood test indices (LY, MONO, PLT, MPV, MPV/PC) and inflammatory markers (PCT) were significantly different between groups (p < 0.05). Additionally, it was found that non survival group had less time to develop AB-sBSI (4.000 *vs* 13.000, p<0.001).

**Table 4:** Comparisons of baseline characteristics of AB-sBSI patients between Non survival and survival.

Variables	Non survival (*n* = 39)	Survival (*n* = 31)	*p*-value
Male	31 (79.5%)	23 (74.2%)	0.761
Age (year)	54.615 ± 18.613	53.194 ± 20.827	0.764
Day of hospitalization (day)	18.000(12.750-29.250)	59.000(38.500-89.500)	<0.001 ^**^
Day of ICU hospitalization (day)	14.000(8.000-23.500)	37.000(17.000-74.500)	0.009 ^**^
Temperature (°C)	36.992 ± 0.853	37.129 ± 1.039	0.547
APACHE Ⅱ score	20.758 ± 4.555	16.815 ± 5.664	0.004 ^**^
Laboratory findings			
WBC (x10^9^/L)	10.810(5.335-15.082)	11.220(9.440-13.920)	0.605
LY (x10^9^/L)	0.713 ± 0.462	1.185 ± 0.553	<0.001 ^**^
MONO (x10^9^/L)	0.462 ± 0.407	0.654 ± 0.321	0.037 ^*^
RDW (%)	1.537 ± 4.843	2.672 ± 5.874	0.379
PLT (x10^9^/L)	126.905 ± 102.120	206.164 ± 102.808	0.002 ^**^
MPV (fl)	12.175(11.485-13.805)	10.690(10.070-11.970)	0.004 ^**^
MPV/PC (fl/10^9^/L)	0.110(0.065-0.215)	0.060(0.040-0.080)	0.004 ^**^
CRP (mg/L)	110.953 ± 73.786	91.029 ± 57.449	0.355
ALB (g/L)	32.033 ± 4.842	34.450 ± 4.937	0.094
PCT (ng/mL)	3.530(1.710-18.690)	1.115(0.532-5.008)	0.008 ^**^
ProBNP (pg/mL)	3205.00(1156.00-8610.00)	1457.77(738.70-3508.50)	0.085
Days from the first isolation of AB in non-blood site to blood site	4.000(3.000-9.000)	13.000(6.000-25.500)	<0.001 ^**^

*P<0.05;**P<0.01.

APACHE II, The Acute Physiology and Chronic Health Evaluation II; WBC, leucocyte count; NEU, Neutrophil count; LY, Lymphocyte count; MONO, Monocyte count; RDW, Erythrocyte hemoglobin distribution width; PLT, platelet count; MPV, mean platelet volume; MPV/PC, mean platelet volume/platelet count; CRP, C reactive protein; ALB, albumin; PCT, procalcitonin; ProBNP, Pterior-brain natriuretic peptide.

Cox regression analysis indicated that the number of hospitalization days, NLR, MPV/PC, and the time interval from the initial isolation of AB bacteria in a non-blood site to the occurrence of AB-sBSI were significantly associated with the 30-day mortality rate among patients diagnosed with AB-sBSI (*p* < 0.05) (**Table 5**).

**Table 5:** Cox regression analysis of between non-survival and survival in patients with AB-sBSI.

Variables	Univariable analysis			Multivariable analysis		
	*HR*	*HR* (95%*CI*)	*p*-value	*RR*	*RR* (95%*CI*)	*p*-value
Day of hospitalization (day)	0.919	0.885-0.954	0.000 ^**^	0.937	0.894-0.983	0.008 ^**^
Day of ICU hospitalization (day)	1.000	0.996-1.003	0.806			
APACHE Ⅱ score	1.071	1.006-1.140	0.031 ^*^			
LY	0.114	0.043-0.301	0.000 ^**^			
MONO	0.211	0.065-0.680	0.009 ^**^			
PLT	0.993	0.989-0.997	0.001 ^**^			
NLR	1.035	1.021-1.048	0.000 ^**^	1.022	1.005-1.039	0.011 ^*^
MPV	1.007	0.996-1.019	0.209			
MPV/PC	465.327	37.371-5794.118	0.000 ^**^	1608.562	11.782-219609.852	0.003 ^**^
PCT	1.022	1.007-1.037	0.004 ^**^			
Days from non-blood to blood site	0.887	0.838-0.940	0.000 ^**^	0.863	0.770-0.966	0.010 ^*^

APACHE II, The Acute Physiology and Chronic Health Evaluation II; LY, Lymphocyte count; MONO, Monocyte count; PLT, platelet count; NLR, neutrophil-to-lymphocyte ratio; MPV, mean platelet volume; MPV/PC, mean platelet volume/platelet count; PCT, procalcitonin.

#### 3.2.2 Evaluation of different models

**Figure 4:**
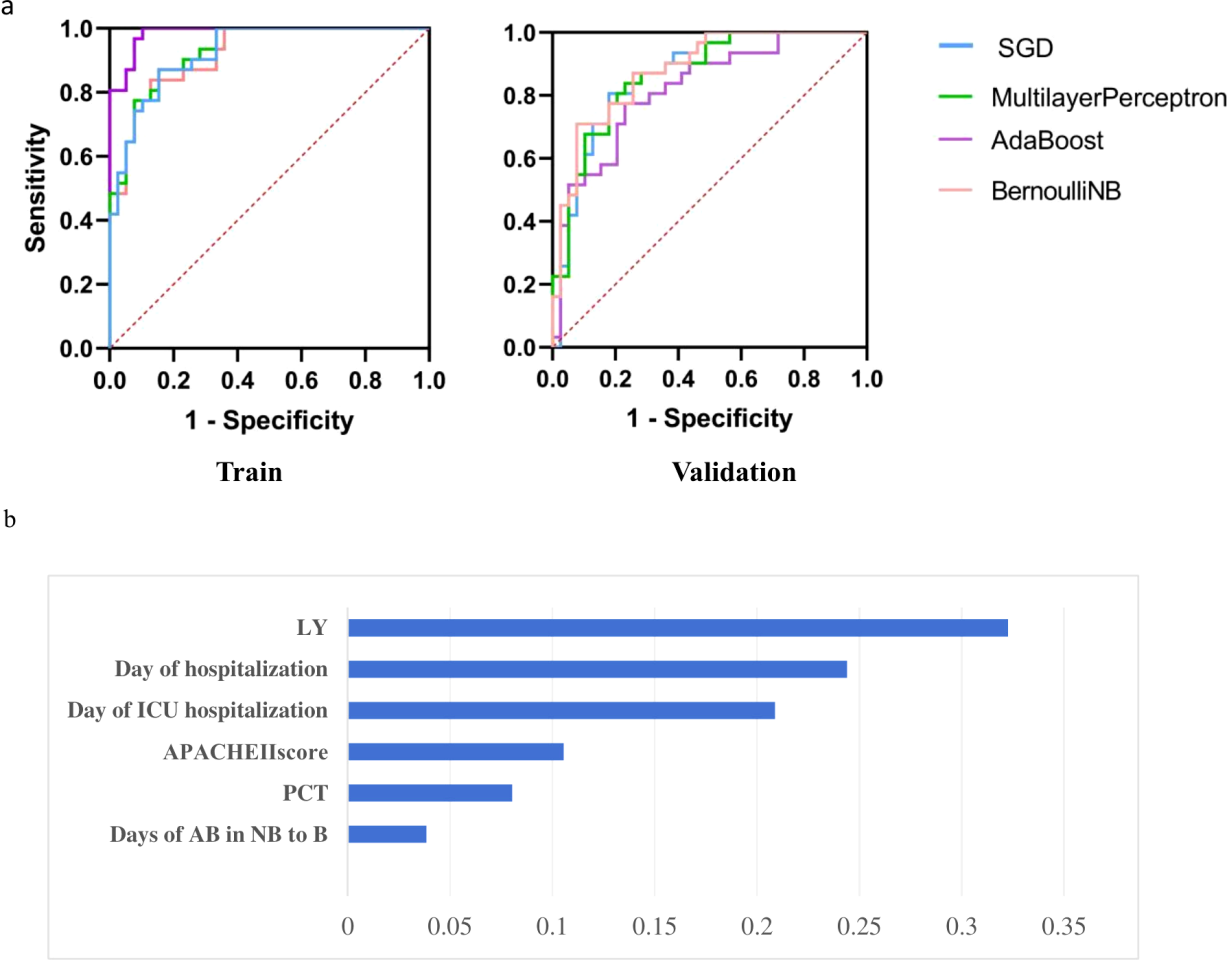
APACHE Ⅱ score, The Acute Physiology and Chronic Health Evaluation II; LY, Lymphocyte count; PCT, Calcitonin; Days of AB in NB to B, Days from the first isolation of AB in non-blood site to blood site. **(A)** The AUROC of Prognosis Prediction performance in the train and validation datasets. **(B)** Feature Importance in AdaBoost model of Prognosis.

Based on the highest AUC of the AdaBoost model in the training set, and its positive difference compared to the other four models (p < 0.01) in **Table 6**, we ultimately selected the AdaBoost model as our AB-sBSI prognostic prediction model. As shown in **Table 7**, the AUROC results of AdaBoost were 0.986 and 0.821 on the train and validation datasets, with acceptable specificity and sensitivity in train dataset (0.949 and 0.839, respectively). **Figure 4a** demonstrated all four models have effective predictive performance in AB-sBSI prognosis. Confusion matrix of AdaBoost model was in the **Supplementary Table S9**. The web application of the model can be access in https://dxonline.deepwise.com/prediction/index.html?baseUrl=%2Fapi%2F&id=49468&topicName=undefined&from=share&platformType=wisdom. A screenshot of the webpage was shown in **Supplementary Image 1**. Upon inputting the required parameters, the system can determine the likelihood of a patient having either non-survival and survival.

**Table 6:** Delong test between four models in prognostic performance.

Delong test between four models	Training Delong		Validation Delong	
Models	z	p	z	p
AdaBoost-SGD	2.51	0.012 *	-1.072	0.284
AdaBoost-MultilayerPerceptron	2.44	0.015 *	-1.194	0.232
AdaBoost-BernoulliNB	2.731	0.006 **	-1.85	0.064
SGD-MultilayerPerceptron	-1.263	0.206	-0.147	0.883
SGD-BernoulliNB	0.313	0.754	-0.526	0.599
MultilayerPerceptron-BernoulliNB	0.652	0.514	-0.524	0.6

**Table 7:** Prognosis prediction performance of the outcome of patients with AB-sBSI.

Dataset	ML models	AUROC	Sensitivity	Specificity	Negative predictive value	Positive predictive value
Train dataset	AdaBoost	0.986 (0.968-1.000)	0.839 (0.681-0.932)	0.949 (0.826-0.990)	0.881 (0.757-0.951)	0.929 (0.788-0.984)
	MultilayerPerceptron	0.931 (0.877-0.985)	0.806 (0.637-0.911)	0.872 (0.723-0.950)	0.850 (0.701-0.935)	0.833 (0.668-0.932)
	BernoulliNB	0.921(0.861-0.980)	0.839 (0.681-0.932)	0.821 (0.657-0.922)	0.865 (0.705-0.949)	0.788 (0.621-0.900)
	SGD	0.926 (0.868-0.983)	0.742 (0.568-0.868)	0.923 (0.792-0.978)	0.818 (0.669-0.914)	0.885 (0.724-0.961)
Validation dataset	BernoulliNB	0.881 (0.803-0.959)	0.806 (0.647-0.910)	0.743 (0.574-0.867)	0.829 (0.666-0.926)	0.714 (0.550-0.840)
	MultilayerPerceptron	0.867(0.784-0.950)	0.806 (0.647-0.910)	0.795 (0.636-0.901)	0.838 (0.681-0.930)	0.758 (0.595-0.874)
	SGD	0.865(0.779-0.951)	0.710 (0.535-0.842)	0.821 (0.664-0.919)	0.780 (0.627-0.888)	0.759 (0.582-0.885)
	AdaBoost	0.821(0.721-0.920)	0.677 (0.504-0.816)	0.795 (0.636-0.901)	0.756 (0.598-0.871)	0.724 (0.543-0.858)

#### 3.2.3 Clinical features importance

**Figure 4b** shows the top six significance values of the selected laboratory tests in AdaBoost model. Each of these six tests exhibited an importance greater than 0.2, with LY having the utmost weight. This was followed by Days of hospitalization, Days of ICU hospitalization, APACHE II score, PCT and Days from the first isolation of AB in non-blood site to blood site, in order of significance.

#### 3.2.4 Comparison of the AdaBoost model with APACHE II and NLR

We conducted a comparative analysis of the predictive performance between the AdaBoost model and the use of APACHE II and NLR alone, which are the important prevalent biomarkers for prognostic prediction in clinical practice (as shown in **Figure 3b**) (Liang and Yu, 2022). The findings revealed that the AdaBoost model outperformed APACHE II and NLR (AUROC 0.972 *vs*. AUROC 0.700 and 0.703).

## 4 Discussion

In this study, we established a diagnostic model for predicting the risk of AB-sBSI in ICU patients and a prognostic model for assessing the associated 30-day mortality risk. These models have been converted into web-based tools to provide clinicians with actionable insights for rapidly adjusting treatment strategies. In line with prior evidence that incorporating overlooked variables can improve predictive accuracy beyond standard models, this study incorporated underutilized clinical variables—such as time-related metrics, invasive mechanical ventilation, and catheterization—to enhance performance (Zhou et al., 2025).

In this study, the AB-sBSI risk model outperformed competing models for both diagnosis and 30-day mortality, as well as demonstrated higher predictive accuracy than established markers such as C-reactive protein, APACHE II, and NLR. The superior performance of AdaBoost can be attributed to its intrinsic ensemble learning mechanism. Unlike linear models such as Linear Discriminant Analysis and Logistic Regression, AdaBoost iteratively refines its predictions by focusing on misclassified instances through sample weight adjustments. This adaptive nature allows it to effectively capture the complex, nonlinear interactions among heterogeneous clinical variables characteristic of the ICU setting (Christodoulou et al., 2019; Henry Basil et al., 2024). The markedly higher AUC observed with AdaBoost underscores the advantage of this approach in modeling the multifaceted pathophysiology of AB-sBSI. Furthermore, feature importance analysis visually identified key clinical predictors, reinforcing that the model’s decisions are driven by a composite of clinically relevant variables rather than relying on a single biomarker.

Given that ICU patients are often critically ill with compromised immunity, AB-sBSI portends a poor prognosis. Early identification of sBSI risk in AB-colonized patients is crucial, as it opens a critical window for initiating targeted anti-infective therapies (Gouel-Cheron et al., 2022). While recent studies have advanced the development of pragmatic diagnostic models for drug-resistant infections—such as an XDRAB risk model with Lasso regression (Shi et al., 2024) and a high-performing hybrid random forest model for ICU multi-drug resistant bacteria (He and Qu, 2023)—they have not specifically addressed the prediction of sBSI in the ICU setting (Roimi et al., 2020). This gap underscores the distinct focus and novelty of the present study.

In the diagnostic model for predicting the risk of AB-sBSI in ICU patients, by leveraging readily accessible clinical indicators, this model enables early identification of sBSI risk following AB isolation, thereby facilitating timely infection control and treatment optimization. Notably, the finding that RDW and MPV were the influential predictors in our model provides critical insights. From a pathophysiological perspective, infection-related oxidative stress is known to inhibit erythrocyte precursor maturation and promote premature release of immature cells into circulation—a mechanism that may explain the elevated RDW values observed in AB-sBSI patients (Mahmood et al., 2014). In the setting of BSI, pro inflammatory cytokines (e.g., IL-6, TNF-α) disrupt iron metabolism and erythropoiesis, leading to the release of immature, variably sized red cells and an increase in RDW (Jin et al., 2025). Previous studies have shown that an elevated RDW on admission independently predicts mortality, length of stay, and the risk of subsequent bloodstream infection in critically ill patients (Tseng et al., 2020). Accordingly, considering both statistical significance and clinical applicability, we incorporated RDW as a variable in the design of our models. The prominence of tracheal intubation/tracheotomy as a predictor underscores its role in AB-sBSI risk. This intervention compromises natural airway defenses (Zilberberg et al., 2016; de Carvalho Baptista et al., 2018), consistent with its established association with infection (Zoabi et al., 2021; Choi et al., 2024).

Furthermore, ML further identified modifiable ICU-related predictors. Although these relationships are correlational, they suggest that reducing ICU stay and optimizing airway management could help mitigate AB-sBSI risk. Future studies could adopt a target-trial emulation (TTE) framework to integrate predictive modeling with causal inference, enabling rigorous evaluation of these factors (Yang et al., 2025).

The 30-day mortality risk model leverages explainable AI to provide interpretable clinical decision support. A crucial finding is the significantly shorter transition time from non-blood AB isolation to bacteremia in non-survivors. This abbreviated interval likely reflects greater disease severity, potentially due to factors such as initial inappropriate antibiotic therapy or more frequent invasive procedures (Ju et al., 2018). Moreover, this parameter likely encapsulates the dynamic interplay between bacterial virulence and compromised host defenses, offering critical insights for timely intervention (Holmes et al., 2024).

Moreover, the significantly higher LY counts observed in the survival group suggested that LY may serves as a surrogate for adaptive immune competence. BSI induced lymphocyte apoptosis and functional exhaustion result in lymphopenia, which correlates with impaired pathogen clearance, higher rates of secondary infection, and increased organ dysfunction (Yang et al., 2015). The clinical accessibility of LY counts within routine complete blood count (CBC) parameters, combined with this mechanistic rationale, supported its inclusion in our model.

Although our findings confirm that the NLR is an independent risk factor for mortality—consistent with prior studies (Huang et al., 2024; Jin et al., 2024; Wei et al., 2024)—it was ranked below six other variables in feature importance. This discrepancy highlights the methodological distinction between Cox regression, which identifies statistically significant associations with outcome at the cohort level, and machine learning feature importance, which reflects a variable’s contribution to individual-level prediction accuracy (Qiu et al., 2020; Sharafi et al., 2024). NLR may represent a universal risk factor with limited interpatient variability, thus offering high epidemiological relevance but lower discriminative power in a multivariable predictive model.

This study was conducted in a multicenter ICU cohort with inherent clinical heterogeneity. While our model provides a general predictive framework, the importance of specific predictors may vary across patient subgroups (e.g., surgical *vs*. medical). Future work should therefore pursue subgroup-specific analyses to support more personalized prognosis (Yang et al., 2024).

This study has several limitations. First, despite being a multi-center dataset, the sample size was moderate, and external validation was limited, which restricts the model’s stability and generalizability. Second, the retrospective design is inherently susceptible to selection bias and likely underrepresents milder cases. Third, although internal validation methods were applied, the model’s robustness may be compromised by the suboptimal predictor-to-sample ratio and the mean imputation for handling missing data. Therefore, independent external validation is mandated prior to any clinical implementation.

## 5 Conclusion

We have developed two readily accessible online prediction tools: an AB-sBSI Risk Prediction Diagnostic model and a 30-day mortality risk prediction model for ICU patients. Designed to support clinical decision-making, these tools provide a foundation for individualized treatment strategies. As an initial exploratory step, they hold potential to aid in improving patient outcomes and guiding infection prevention and control measures in the ICU. Further validation across diverse populations and healthcare settings will be essential to confirm their broader applicability.

## Data Availability

The datasets used and/or analyzed during the current study available from the corresponding author on reasonable request. Requests to access the datasets should be directed to chenyli3@mail.sysu.edu.cn.
