# Supplementary material for: Early diagnosis and prognostic prediction of secondary bloodstream infections caused by Acinetobacter baumannii in critically ill patients by machine-learning algorithms
Source: Front Cell Infect Microbiol. 2026 Jan 8;15:1667176. doi: 10.3389/fcimb.2025.1667176 (PMC12823858; doi:10.3389/fcimb.2025.1667176)

Decision Analysis and Calibration plots of the AB-sBSI Risk Prediction Diagnostic Model (Adaboost)


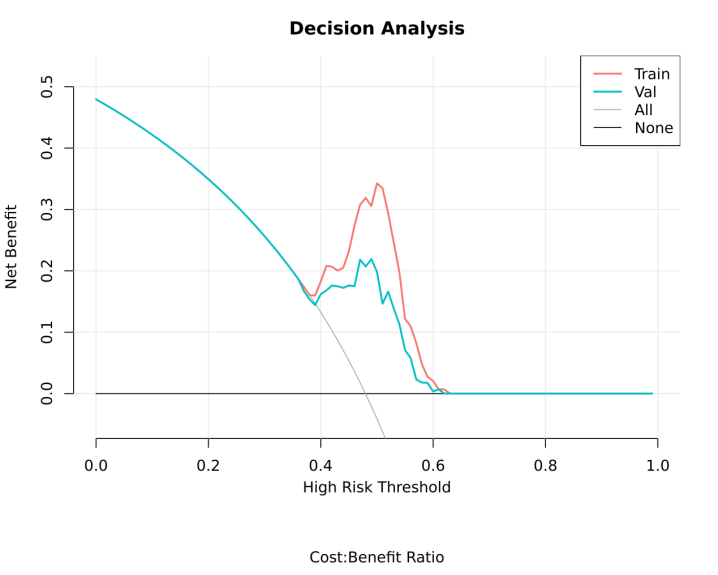


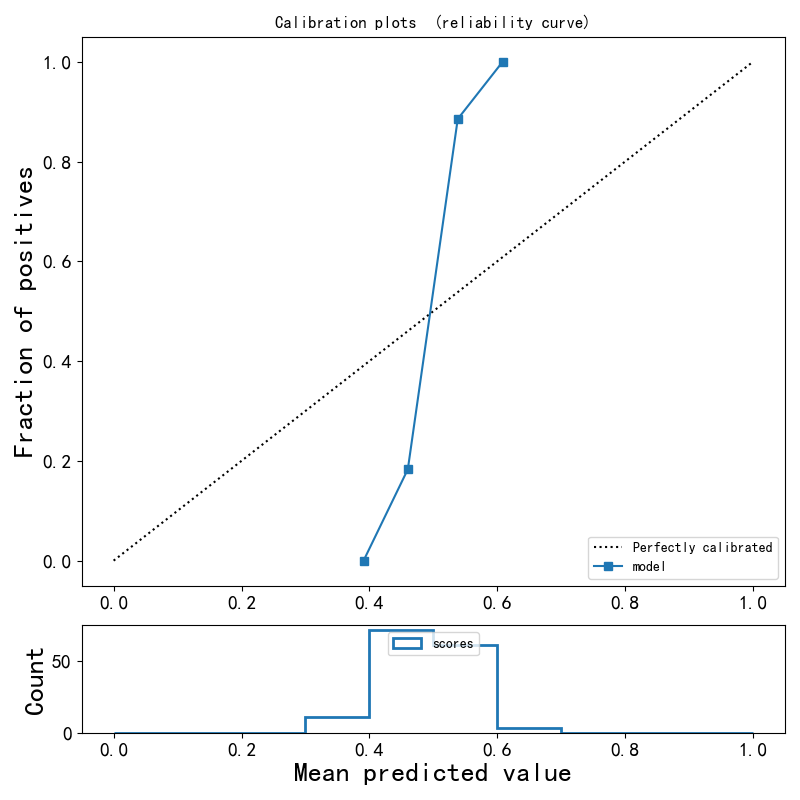

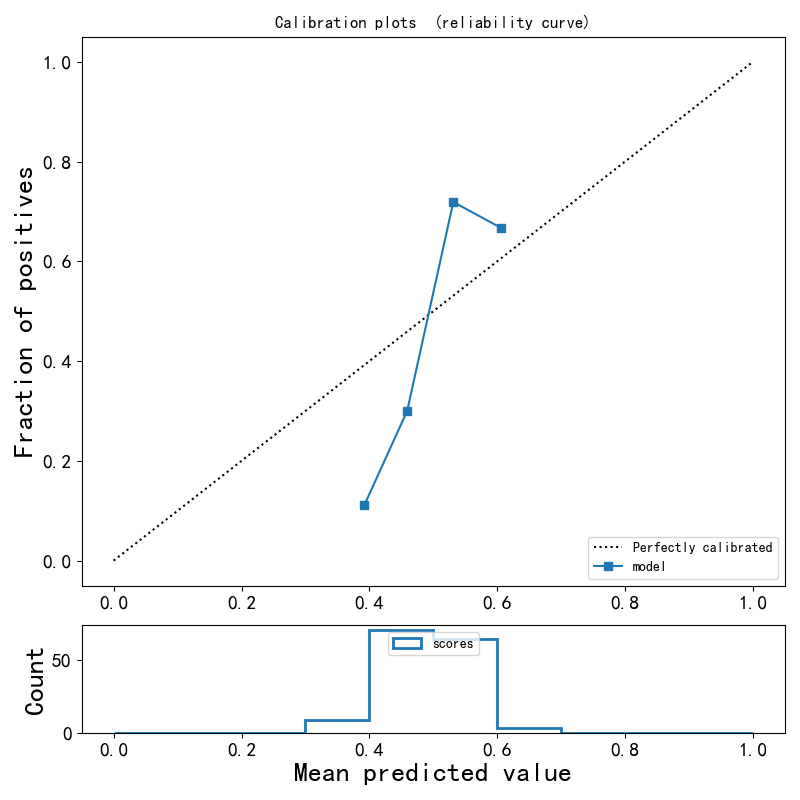


Decision Analysis and Calibration plots of the AB-sBSI Risk Prediction Diagnostic Model (LinearDiscriminantAnalysis)


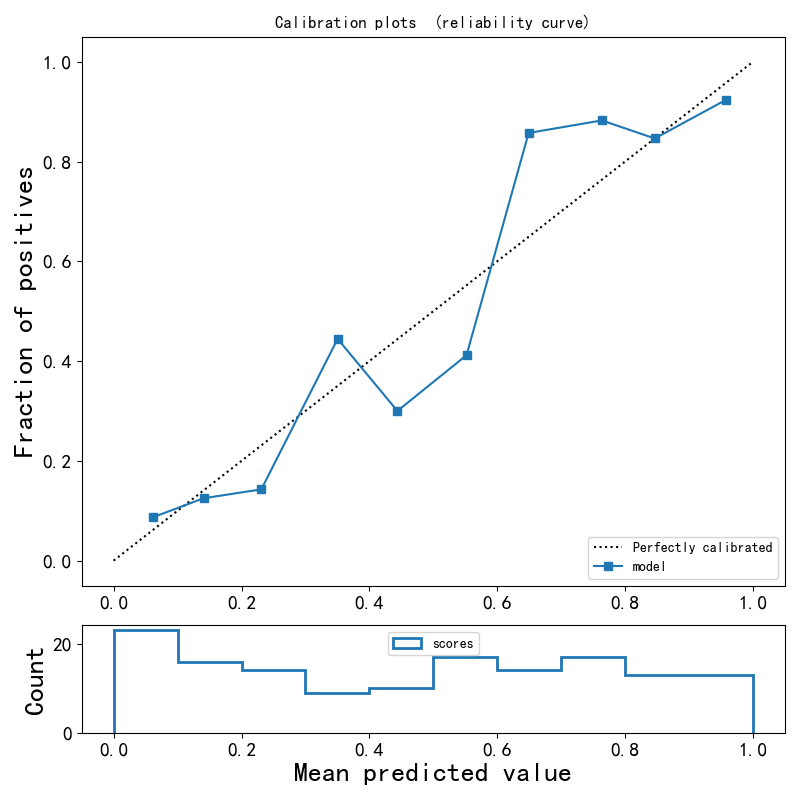

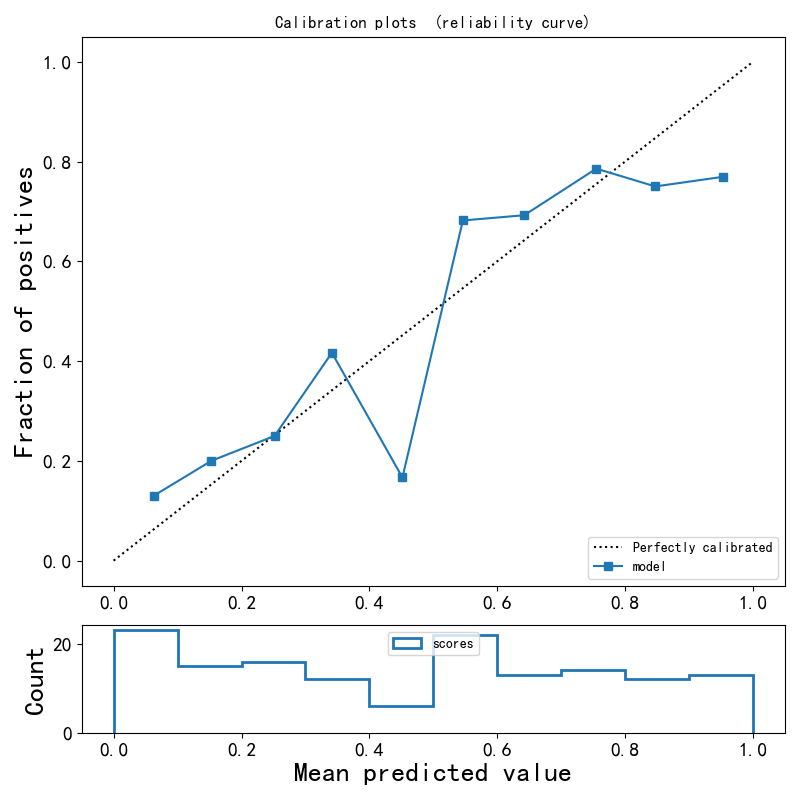

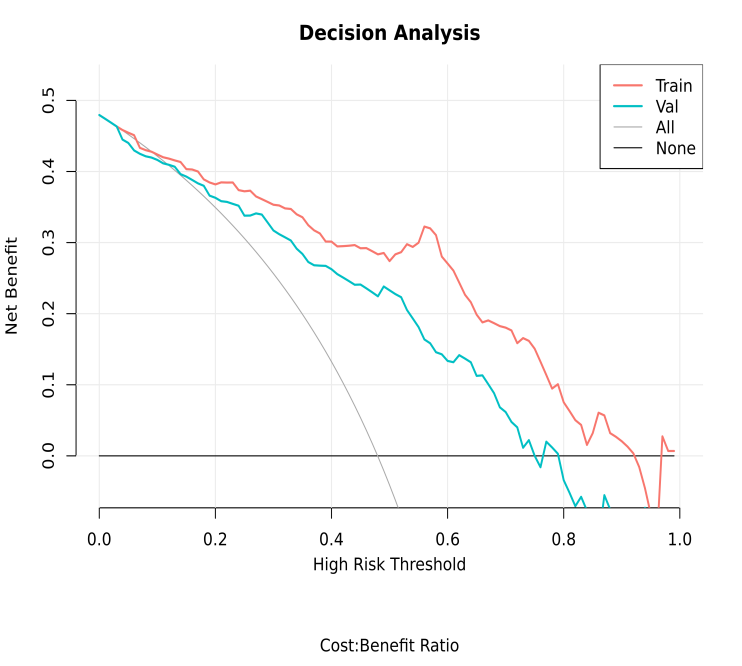


Decision Analysis and Calibration plots of the AB-sBSI Risk Prediction Diagnostic Model (LogisticRegression)


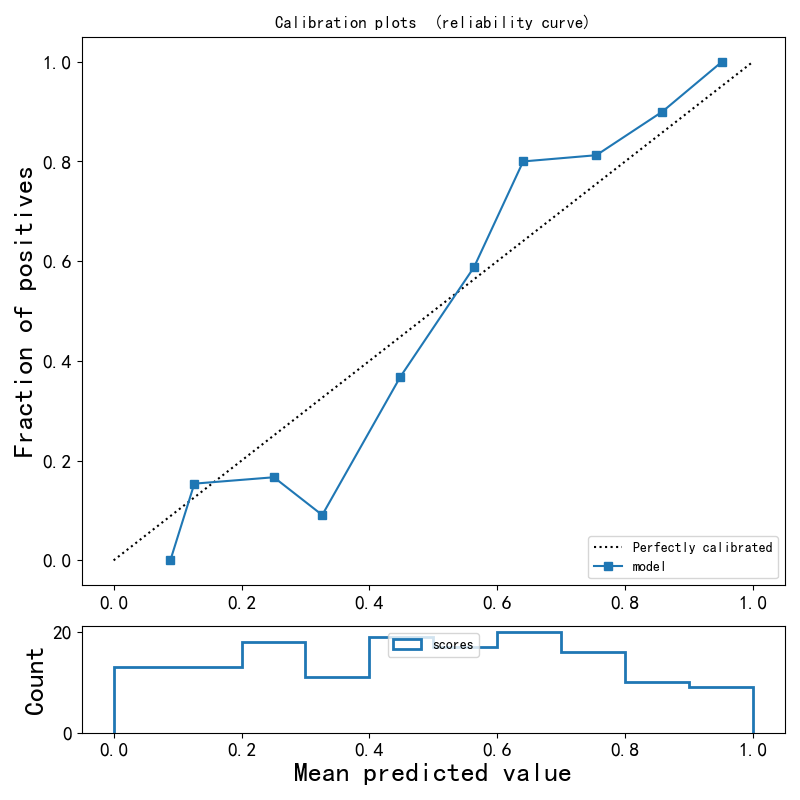

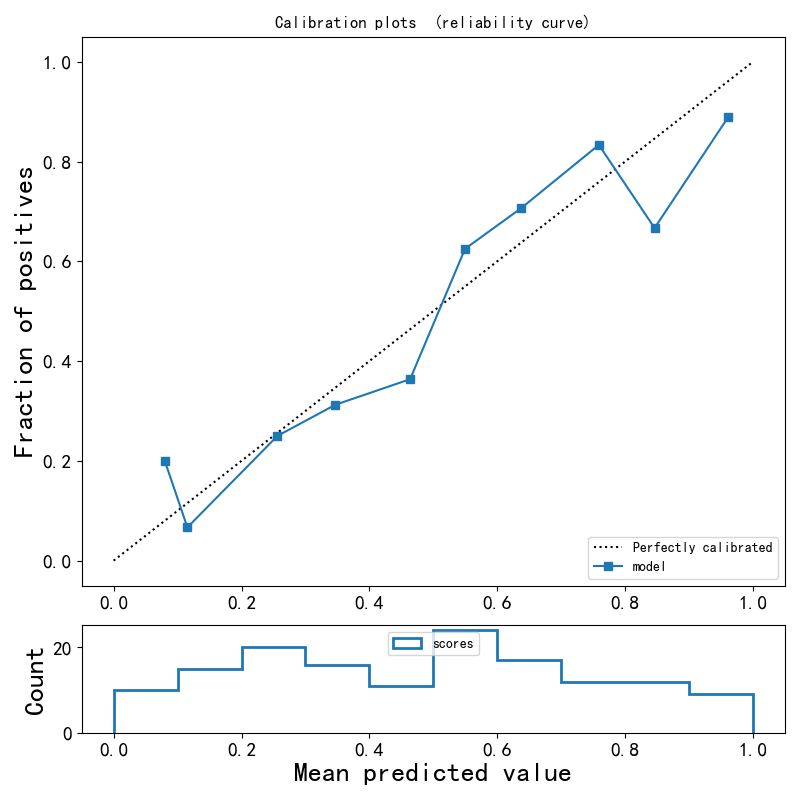

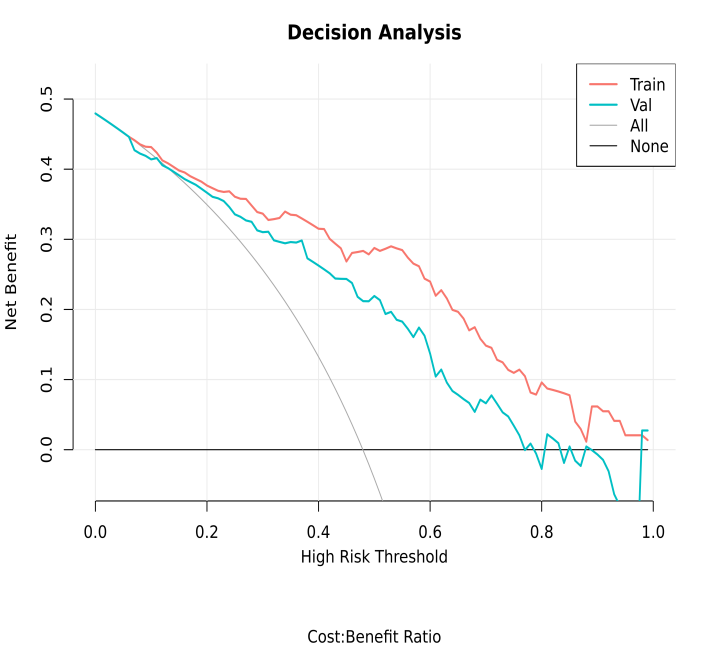

Supplement: Supplementary file 1 [file DataSheet1.docx]
