# Supplementary figures and images for: Early diagnosis and prognostic prediction of secondary bloodstream infections caused by Acinetobacter baumannii in critically ill patients by machine-learning algorithms
Source: Front Cell Infect Microbiol. 2026 Jan 8;15:1667176. doi: 10.3389/fcimb.2025.1667176 (PMC12823858; doi:10.3389/fcimb.2025.1667176)

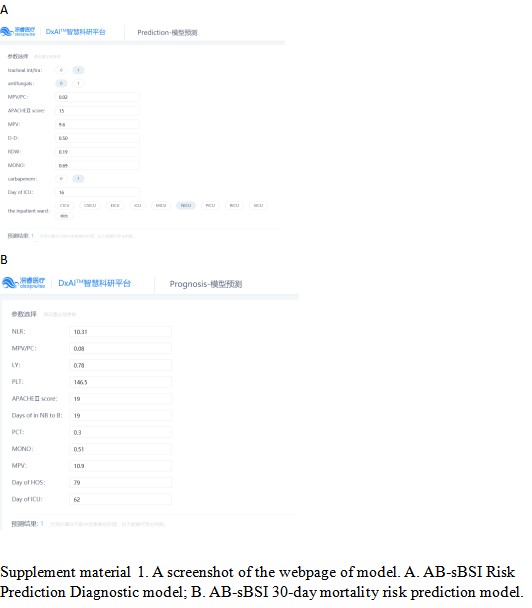

Supplement: Supplementary file 3 [file Image1.jpeg]
